# Drug-Related Pyroglutamic Acidosis: Systematic Literature Review

**DOI:** 10.3390/jcm13195781

**Published:** 2024-09-27

**Authors:** Tessa Scafetta, Orsolya Kovacs, Gregorio P. Milani, Gabriel Bronz, Sebastiano A. G. Lava, Céline Betti, Federica Vanoni, Mario G. Bianchetti, Pietro B. Faré, Pietro Camozzi

**Affiliations:** 1 Family Medicine Institue, Faculty of Biomedical Sciences, Università della Svizzera Italiana, 6900 Lugano, Switzerland; tessa.scafetta@eoc.ch (T.S.); orsolya.kovacs@hopitalvs.ch (O.K.); gabriel.bronz@eoc.ch (G.B.); pietro.fare@usz.ch (P.B.F.); 2 Department of Anesthesia, Hôpital du Valais, 1951 Sion, Switzerland; camozzip@gmail.com; 3 Pediatric Unit, Fondazione IRCCS Ca’ Granda Ospedale Maggiore Policlinico, 20122 Milan, Italy; gregorio.milani@unimi.it; 4 Department of Clinical Sciences and Community Health, Università degli Studi di Milano, 20122 Milan, Italy; 5 Pediatric Cardiology Unit, Department of Pediatrics, Centre Hospitalier Universitaire Vaudois, University of Lausanne, 1011 Lausanne, Switzerland; webmaster@sebastianolava.ch; 6 Clinical Pharmacology Service, Centre Hospitalier Universitaire Vaudois, University of Lausanne, 1011 Lausanne, Switzerland; 7 Pediatric Emergency Department, University Children’s Hospital Zurich, 8032 Zurich, Switzerland; 8 Pediatric Institute of Southern Switzerland, Ente Ospedaliero Cantonale, 6500 Bellinzona, Switzerland; federica.vanoni@eoc.ch; 9 Faculty of Biomedical Sciences, Università Della Svizzera Italiana, 6900 Lugano, Switzerland

**Keywords:** acetaminophen, vigabatrin, β-lactamase-resistant penicillin, acid base equilibrium, drug-related side effect, 5-oxoproline

## Abstract

**Background**: Inborn errors of glutathione metabolism may cause high anion gap metabolic acidosis due to pyroglutamic acid accumulation. Since 1988, cases of this acidosis have been reported in individuals without these defects. **Methods**: Given the poorly characterized predisposing factors, presentation, management, and prognosis of acquired pyroglutamic acidosis, we conducted a systematic review using the National Library of Medicine, Excerpta Medica, Web of Science, and Google Scholar databases. **Results**: A total of 131 cases were found. Most patients were females (79%), adults (92%) aged 51 years or older (66%) with pre-existing conditions (74%) such as undernutrition, alcohol-use disorder, or kidney disease, and had an ongoing infection (69%). The clinical features included diminished consciousness (60%), Kussmaul breathing (56%), and nausea or vomiting (27%). At least 92% of patients were on paracetamol therapy for >10 days at an appropriate dose, 32% on a β-lactamase-resistant penicillin, and 2.3% on vigabatrin. Besides severe anion gap acidosis, patients also presented with hypokalemia (24%) and kidney function deterioration (41%). Management involved discontinuing the offending drug (100%), bicarbonate (63%), acetylcysteine (42%), and acute kidney replacement therapy (18%). The fatality rate was 18%, which was higher without acetylcysteine (24%) compared to with it (11%). **Conclusions**: Acquired pyroglutamic acidosis is a rare, potentially fatal metabolic derangement, which usually occurs after paracetamol use, frequently combined with a β-lactamase-resistant penicillin or vigabatrin. This condition predominantly affects adults, especially women with factors like undernutrition, alcohol-use disorder, or kidney disease, often during infection. Increased awareness of this rare condition is necessary.

## 1. Introduction

The accumulation of organic acids is a well-known cause of metabolic acidosis [1]. This acid–base imbalance often arises in conditions that lead to increased levels of L-lactic acid or ketones (primarily β-hydroxybutyric acid), and in advanced kidney disease [1]. Other contributors include the production of D-lactic acid by gut bacteria [2] and the consumption or administration of (di)-ethylene glycol, methanol, or propylene glycol [1]. Finally, metabolic acidosis can also result from an accumulation of acids in newborns and infants affected by an inherited defect in the metabolism of organic acids, pyruvate, ketones, and glutathione [3].

Inborn errors of glutathione metabolism may induce acidosis due to the accumulation of pyroglutamic acid, also referred to as 5-oxoproline [3]. Since 1988, cases of pyroglutamic acidosis have been reported in infants and children without any inborn defect of glutathione metabolism [4]. An association of this acquired form of acidosis was proposed with female sex, undernutrition, chronic alcohol-use disorder, pre-existing kidney disease, and the use of drugs such as vigabatrin, β-lactamase-resistant penicillin, and especially paracetamol [4,5,6,7].

Although individual case reports offer valuable insights into extraordinarily unusual conditions, only their cumulative analysis through systematic reviews can reveal patterns, clarify clinical features, and highlight effective treatment approaches that may not be apparent from isolated reports. As a result, such systematic reviews can lead to improved patient care, more informed clinical decision making, and the identification of key areas for future research, ultimately advancing knowledge in the field [8]. To gain a better understanding of drug-related pyroglutamic acidosis, we performed a systematic review of the literature [8].

## 2. Materials and Methods

### 2.1. Registration—Bibliographic Search

Approval from an Institutional Review Board was not necessary for this literature review. The investigation [8] was registered at the International Prospective Register of Systematic Reviews (PROSPERO: CRD42024520897) and carried out in accordance with the last edition of the Preferred Reporting Items for Systematic Reviews and Meta-Analyses guidelines [9]. The databases utilized for this study were Excerpta Medica, United States National Library of Medicine, and Web of Science, with no restrictions on language or publication date [8]. The search methodology involved using the following combinations of terms: (acidosis OR anion gap OR pyroglutamic acid OR 5-oxoproline) AND (acetaminophen OR paracetamol OR penicillinase-fast penicillin OR β-lactamase-resistant penicillin OR flucloxacillin OR vigabatrin). Relevant articles cited in the obtained documents, as well as the literature available on Google Scholar, were also evaluated for possible inclusion [10]. The initial search was conducted in April 2024, with a subsequent update prior to manuscript submission. After a preliminary screening of titles and abstracts, the full texts of the chosen reports were evaluated for eligibility.

### 2.2. Selection Criteria—Data Extraction—Definitions

Original reports documenting individual humans with an acquired pyroglutamic acidosis (Table 1) were considered. The diagnosis was established in subjects displaying an otherwise unexplained metabolic acidosis, not fully accounted for by the accumulation of L-lactic acid or ketones. Patients with a history indicative of D-lactic acidosis or acidosis related to (di)-ethylene glycol, methanol, or propylene glycol were excluded [1,2,3]. The diagnosis was further supported by a relevantly elevated blood anion gap or a strongly positive pyroglutamic acid test in either urine or blood [1,4]. The following eight variables were extracted for each individual case using a pilot-tested checklist: (1) demographics and pre-existing conditions with an emphasis on undernutrition, chronic alcohol-use disorder, kidney disease, or pregnancy; (2) acute, recurrent, or chronic infections likely of bacterial origin; (3) existing medication with drugs such as paracetamol, β-lactamase-resistant penicillin, or vigabatrin [5,6,7]; (4) clinical features with an emphasis on a diminished level of consciousness, Kussmaul breathing [11], and nausea or vomiting; (5) laboratory features with an emphasis on acid–base balance, sodium, potassium, chloride, lactic acid, kidney function, liver parameters, ammonia or hemoglobin in blood, and the urinary Rothera nitroprusside-glycine dipstick test for ketone bodies; (6) tests for pyroglutamic acid; (7) drug management with an emphasis on sodium bicarbonate and acetylcysteine; and (8) recurrences and outcome. Case series exploring the possible interaction between acid–base balance and pyroglutamic acid were also considered.

The diagnostic criteria described in Table 1 were used to define undernutrition [12], pathologically increased blood anion gap [1], L-hyperlactacidemia, acute kidney function deterioration [13], altered liver parameters [14], hyperammonemia, and anemia.

Two authors independently carried out a duplicate literature search, selected reports for inclusion, and extracted the data. Any discrepancies were resolved through consensus, with a third researcher consulted when necessary. One author entered the information into a pilot-tested sheet, while another verified the accuracy of the data entry.

### 2.3. Reporting Thoroughness—Analysis

For each individual case, the reporting thoroughness of the eight specified variables was evaluated using a 0, 1, or 2 scale. The overall reporting thoroughness of each case was then classified as excellent (14 or higher), good (11 to 13), or satisfactory (8 to 10), based on the total score. The missing data were addressed through pairwise deletion [15]. The categorical data were shown as frequencies and assessed using the Fisher exact test [16]. The continuous data were displayed as median and interquartile range and analyzed using the Kruskal–Wallis H-test [16]. The statistical significance was determined by two-sided *p*-values < 0.05. GraphPad Prism 10.2.3 (GraphPad Software, San Diego, CA, USA) was used for analyses.

## 3. Results

### 3.1. Search Outputs

**Figure 1:**
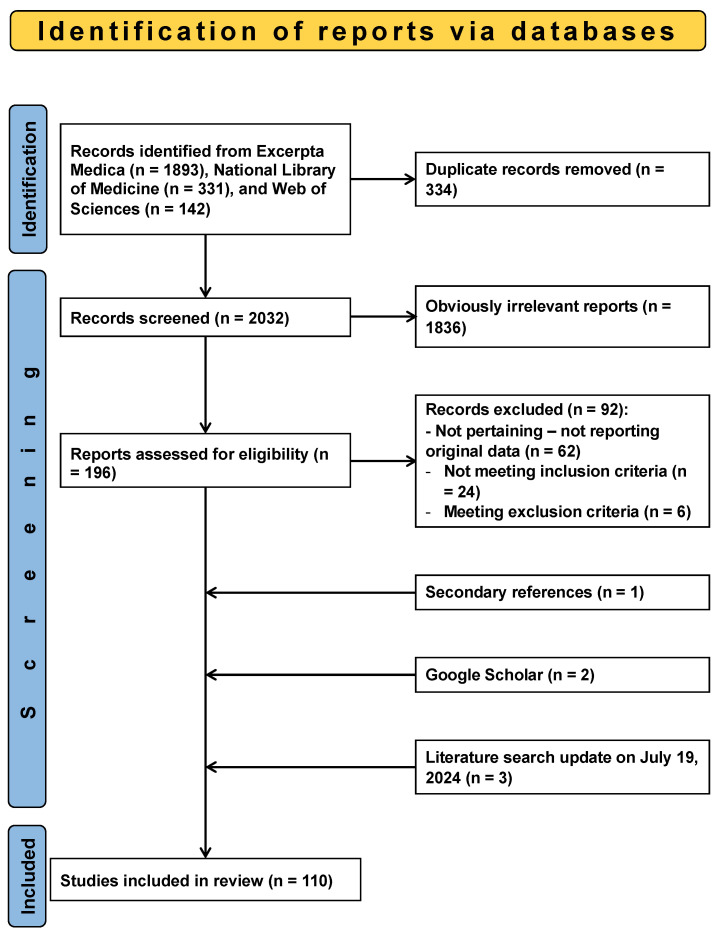
Drug-related pyroglutamic acidosis. Flowchart of the literature search.

The search for the literature yielded 2366 potentially relevant articles (Figure 1).

The literature search process is depicted in Figure 1. As of the latest update on 19 July 2024, 110 reports were included in the final analysis, spanning from 1988 onwards [17,18,19,20,21,22,23,24,25,26,27,28,29,30,31,32,33,34,35,36,37,38,39,40,41,42,43,44,45,46,47,48,49,50,51,52,53,54,55,56,57,58,59,60,61,62,63,64,65,66,67,68,69,70,71,72,73,74,75,76,77,78,79,80,81,82,83,84,85,86,87,88,89,90,91,92,93,94,95,96,97,98,99,100,101,102,103,104,105,106,107,108,109,110,111,112,113,114,115,116,117,118,119,120,121,122,123,124,125,126]. Among these, 105 reports detailed 131 individual cases of acquired pyroglutamic acidosis [17,18,19,20,21,22,23,24,25,26,27,28,29,30,31,32,33,34,35,36,37,38,39,40,41,42,43,44,45,46,47,48,49,50,51,52,53,54,55,56,57,58,59,60,61,62,63,64,65,66,67,68,69,70,71,72,73,74,75,76,77,78,79,80,81,82,83,84,85,86,87,88,89,90,91,92,93,94,95,96,97,98,99,100,101,102,103,104,105,106,107,108,109,110,111,112,113,114,115,116,117,118,119,120,121]. Two of the mentioned reports, along with five additional case series, addressed the possible interaction between acid–base balance and pyroglutamic acid [73,88,122,123,124,125,126]. Of the 110 reports, 99 were in English, 7 in French, 2 in German, and 1 each in Dutch and Spanish. The regional distribution of the reports was as follows: Europe contributed 50 reports (United Kingdom 14; Netherlands 10; Belgium 8; Ireland 6; France, Germany, and Switzerland 3 each; Spain 2; Portugal 1), America 39 (United States 32; Canada 6; Brazil 1), Oceania contributed 15 (Australia 14; New Zealand 1), and Asia 6 (India 3; Israel 2; Lebanon 1).

### 3.2. Findings

#### 3.2.1. Individual Cases

##### Reporting Thoroughness

The thoroughness of reporting for the 131 patients was rated as excellent in 50 cases (38%), good in 74 cases (56%), and satisfactory in the remaining 7 (5.3%).

##### Baseline Clinical and Laboratory Data

**Figure 2:**
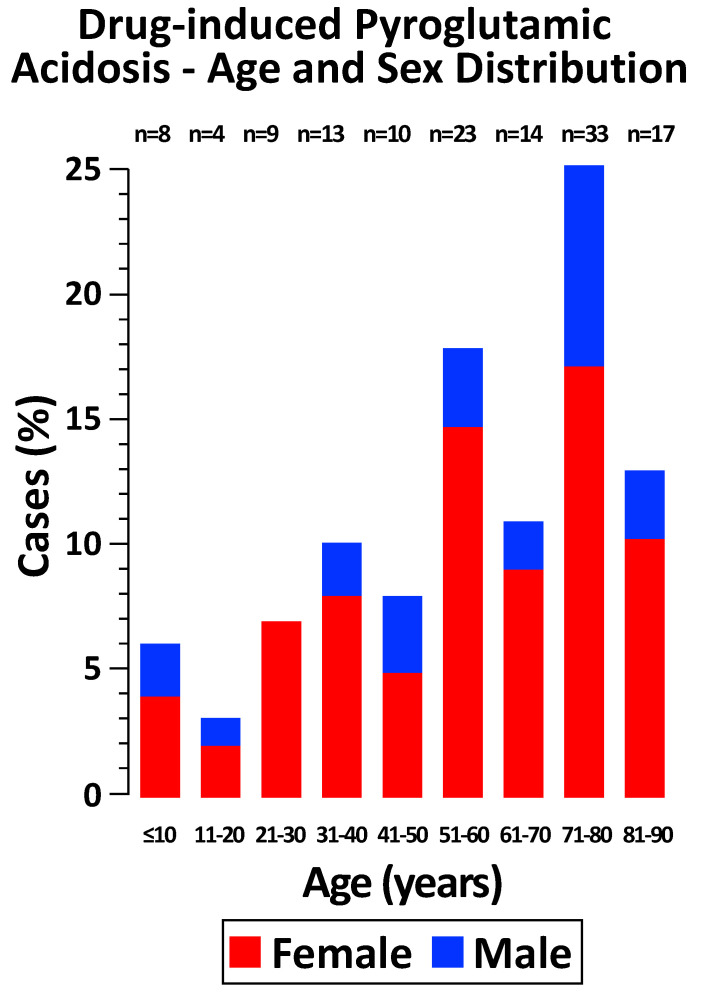
Age and sex distribution of 131 patients with drug-related pyroglutamic acidosis.

Acquired pyroglutamic metabolic acidosis was diagnosed in cases with unexplained metabolic acidosis as previously defined. The pyroglutamic acid test was conducted in 121 individuals, with 115 on urine and 6 on blood samples. Quantitative determinations were performed in 79 instances, while semiquantitative analyses were conducted in the remaining 42 cases. All tests yielded strongly positive results. The anion gap in blood was significantly elevated in all 117 cases where this information was available. Notably, the anion gap showed a marked increase in the 10 cases where the pyroglutamic acid test was not performed. The baseline characteristics of the patients with pyroglutamic acidosis are presented in Table 2 and Figure 2.

Most subjects were female (79%) adults (92%) 51 years or more of age (66%) with a pre-existing condition such as undernutrition, alcohol-use disorder, kidney disease, or pregnancy, and an ongoing infection. The most reported clinical features were diminished consciousness (60%), Kussmaul breathing (56%), and nausea or vomiting (27%). At least 92% of the patients were on therapy with paracetamol, 32% with a β-lactamase-resistant penicillin (usually flucloxacillin), and 2.3% with vigabatrin. A history of recurrent episodes of pyroglutamic acidosis was detected in 6.1% of cases.

The upper panel of Table 3 depicts the dose and duration of paracetamol treatment and the laboratory data for the whole group of patients, for patients uniquely on paracetamol (63%), for patients on paracetamol together with a penicillin (29%) and for the remaining cases (7.6%). In most cases, paracetamol was taken for >10 days (64%) in an appropriate dose (89%).

Interestingly, an inappropriately high dose of paracetamol was more frequently administered in cases where patients were only on this drug (17%) compared to those on both paracetamol and penicillin (2.6%; *p* = 0.0353). In the whole group of 131 patients, a severe anion gap metabolic acidosis (pH 7.19 [7.12–7.29], bicarbonate 7 [5–10] mmol/L, anion gap 25 [20–30] meq/L) with respiratory compensation (pCO_2_ 16 [13–23] mm Hg) was observed. Laboratory features consistent with some degree of L-lactic- or keto-acidosis were detected in approximately one out of every five cases. Hypokalemia (24%) and an acute kidney function deterioration (41%) were also rather common. There were no statistically significant differences in acid–base balance, sodium levels, or the prevalence of acute kidney function deterioration among the three subgroups (paracetamol only, paracetamol and further drugs, and other drugs). The tendency to hypokalemia was more common (*p* = 0.0002) in patients on paracetamol together with penicillin (45%) as compared with patients on paracetamol alone (17%). The blood anion gap was significantly higher by about 5 meq/L (*p* = 0.0489) and the chloride level lower by 8 mmol/L (*p* = 0.0222) in patients on paracetamol alone compared to those on paracetamol together with penicillin. Altered liver parameters, hyperammonemia, and anemia were found in 34%, 6.9%, and 19% of cases, respectively (Table 3).

##### Management and Course

The management and course are depicted in the lower part of Table 2. Paracetamol, the β-lactamase-resistant penicillin or vigabatrin were always discontinued. Sodium bicarbonate and acetylcysteine were prescribed in 63% and 42% of cases, respectively. Sodium bicarbonate was less frequently administered (*p* = 0.0021) in patients on paracetamol alone (55%) as compared with the two remaining subgroups (75%). Following the acute deterioration of kidney function observed in 54 cases, replacement therapy was initiated in 23.

##### Relationship between Drug Therapy and Mortality

Mortality was considerably higher without (18 out of 76 cases; 24%) than with (6 out of 55 cases; 11%) acetylcysteine, but the difference was not statistically (*p* = 0.0707) significant (Table 4).

#### 3.2.2. Case Series

The interplay between acid–base balance and pyroglutamic acid was investigated in seven case series. The results are summarized in Table 5.

## 4. Discussion

The present report compiles data from 131 individuals and seven case series on acquired pyroglutamic acidosis.

This severe and often fatal (about 20%) condition is very rare and mainly (approximately 70% of cases) affects females 51 years or more of age with chronic undernutrition, alcohol-use disorder, pre-existing kidney disease, or during pregnancy. It usually develops in the clinical setting of an ongoing infection, is typically linked to paracetamol (>90% of cases), the most widely used antipyretic and analgesic for mild to moderate pain (such as headaches, muscle aches, toothaches, and arthritis), in a therapeutic dose for over 10 days, sometimes in combination with a β-lactamase-resistant penicillin, and occasionally recurs. The clinical and biochemical data include diminished consciousness, Kussmaul breathing, nausea and vomiting, anion gap acidosis with bicarbonate levels often below 10 mmol/L, hypokalemia, and a frequently severe deterioration of kidney function.

This discussion will focus (1) on the simultaneous presence of other causes of metabolic acidosis; (2) the principles governing the tendency towards hypokalemia; (3) the occurrence of altered liver parameters, hyperammonemia and anemia; (4) the underlying mechanisms (and the management); (5) the sex difference; and (6) the limitations and strengths of this study.

As previously stated, acquired pyroglutamic acidosis usually affects subjects with severe pre-existing conditions such as undernutrition and alcohol-use disorder and with an ongoing infection. Unsurprisingly, therefore, pyroglutamic acidosis was associated with some degree of keto- or lactic-acidosis in approximately every fifth case [1,127,128].

The potassium level was reduced in acquired pyroglutamic acidosis. Hypokalemia is likely caused by undernutrition, alcohol-use disorder, and potassium shifting into cells due to infection-related stress [129]. This electrolyte disturbance was more severe in patients using β-lactamase-resistant penicillin, which are known to increase urinary potassium excretion [130]. Traditionally, metabolic acidosis is assumed to increase blood potassium levels, but this does not occur in lactic or keto-acidosis, and likely also in pyroglutamic acidosis [131].

Alterations in liver parameters and hyperammonemia were observed in approximately every third case. Since most patients had a pre-existing condition, it is difficult to determine whether the mentioned changes were due to the pre-existing illnesses or to paracetamol use. A kidney function deterioration was also frequently observed. Given that many patients had a pre-existing kidney disease, identifying the underlying mechanism is challenging. Hemolytic anemia is common in primary pyroglutamic acidosis [3]. In this analysis, approximately one in five patients was anemic, but the available information did not reveal the underlying causes.

**Figure 3:**
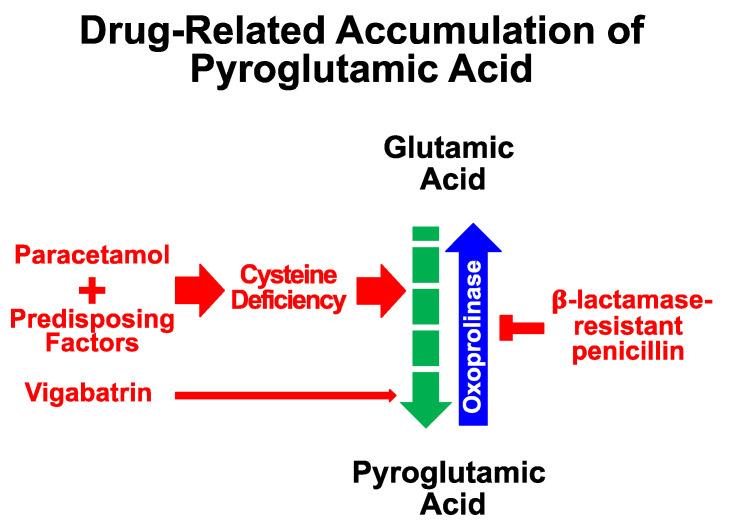
Simplified sketch of the mechanisms underlying the accumulation of pyroglutamic acid on treatment with paracetamol, β-lactamase-resistant penicillin, and vigabatrin. The thickness of the arrows next to paracetamol, flucloxacillin, and vigabatrin is proportional to the relative frequency of the phenomenon.

The aim of this work was not to explore the mechanisms behind acquired pyroglutamic acidosis. A cysteine deficiency is likely crucial in paracetamol-related acidosis [4,132,133]. Simply put, cysteine stores are marginally depleted in females with undernutrition, alcohol-use disorder, kidney disease, and during pregnancy [4,133,134,135,136,137,138]. Paracetamol further depletes cysteine by preventing its recycling [4,133]. This shortage disrupts glutathione synthesis, leading to excess pyroglutamic acid production (Figure 3).

In contrast, an acute liver injury from paracetamol poisoning is due to glutathione depletion, which results in the accumulation of a highly hepatotoxic metabolite [132]. The mechanisms by which β-lactamase-resistant penicillin and vigabatrin may cause pyroglutamic acidosis are speculative. Oxoprolinase, an enzyme in the lactamase family, breaks down pyroglutamic acid into glutamic acid [4]. It is speculated that β-lactamase-resistant penicillin somehow block this pathway (Figure 3), causing an accumulation of pyroglutamic acid. Broad-spectrum β-lactamase inhibitors such as clavulanate may also inhibit oxoprolinase [6,139]. In the present analysis, three cases of paracetamol-related pyroglutamic acidosis occurred in subjects concurrently treated with clavulanate [81,113,118]. Finally, vigabatrin raises ɣ-aminobutyric acid levels and affects the metabolism of glutamic acid, potentially increasing the synthesis of pyroglutamic acid [5].

The treatment of drug-related pyroglutamic acidosis primarily involves discontinuing the causative medication. Administering acetylcysteine, an inexpensive, widely available, and well-tolerated drug that metabolizes into cysteine, is considered a rational approach due to the cysteine deficiency observed in these patients [4,132]. However, our analysis does not conclusively support this strategy. Indeed, evaluating drug effectiveness solely through aggregated individual case data are challenging, and a false negative result due to a limited number of cases is possible. Although drug-related pyroglutamic acidosis is a very rare condition, one might speculate that combining paracetamol with acetylcysteine could be considered, at least in individuals who may have a strong predisposition to this type of metabolic derangement. In many cases, kidney replacement therapy was also applied. Intravenous bicarbonate was frequently prescribed in pyroglutamic acidosis, yet its role in acidosis remains controversial [140].

The mechanisms for the pronounced sex difference in the rates of drug-induced pyroglutamic acidosis are unexplained. We have carefully reviewed the literature and found some interesting information. Many data suggest that the metabolism of glutathione, and consequently cysteine, differs between males and females [141]. Furthermore, the incidence of hyperlactatemia associated with the use of nucleoside analog reverse-transcriptase inhibitors and selective β_2_-adrenoceptor agonists is markedly higher in females than in males [142,143].

This analysis presents both limitations and strengths. The rarity of acquired pyroglutamic acidosis leads to a small sample size. Moreover, the thoroughness of reporting was excellent in no more than 38% of cases, which hinders the power of the evaluation and the generalizability of the results. However, unlike an outstanding recent review [4], this study was registered and conducted following a recognized methodology [9]. Additionally, it employed a comprehensive approach and utilized four databases [4]. Lastly, collaboration with various medical subspecialties enhances its robustness.

This report provides a systematic review of about 130 cases of drug-induced pyroglutamic acidosis published in the literature. The next step could involve gathering and analyzing data from multiple national pharmacovigilance centers. Furthermore, given the scarcity of data on this extremely rare condition, similar to other rare diseases, establishing an expert consensus statement could offer clinicians valuable guidance for diagnosis and treatment.

## 5. Conclusions

Various medications have been linked to elevated levels of lactic acid in the blood. These include, but are not limited to, biguanides, linezolid, propofol, reverse-transcriptase inhibitors, and β_2_-adrenoceptor agonists [143,144]. Additionally, inhibitors of the renal sodium-glucose transporter 2 are emerging as a notable cause of ketoacidosis [129].

Drug-related metabolic acidosis caused by the accumulation of pyroglutamic acid, an intermediate in the glutathione cycle, is a very rare, potentially fatal high anion gap metabolic acidosis. It typically arises after the use of paracetamol (often combined with a β-lactamase-resistant penicillin) for over 10 days. This likely underreported condition mainly affects adults, particularly women with factors like undernutrition, kidney disease, alcohol-use disorder, or pregnancy, often during an ongoing infection. Diagnosis relies on identifying unexplained elevated anion gap acidosis in susceptible individuals. Reaching a definitive diagnosis is challenging because routine laboratory tests do not typically screen for pyroglutamic acid. This specific analysis can only be conducted by specialized central laboratories, limiting the availability of timely diagnostic confirmation.

Healthcare professionals need to be more aware of this rare metabolic condition, which is frequently associated with mild to moderate lactic acidosis or ketoacidosis.

## Figures and Tables

**Table 1:** Definitions used for the present systematic review.

**Metabolic acidosis**: bicarbonate <20 mmol/L, pH < 7.40
**Undernutrition**
blood albumin level ≤ 30 g/L
terms such as “nutritional deficiency”, “stunting”, or “starvation” in the original literature
**Paracetamol dose** per day normal: ≤4000 mg (or ≤75 mg/kg).
**Blood anion gap**
Na^+^ – (Cl^−^ + HCO_3_^−^)
normal: ≤16 meq/L
**L-hyperlactacidemia**
mild: >5.0–10.0 mmol/L
moderate: 10.1–20.0 mmol/L
severe >20.1 mmol/L
**Kidney function deterioration:** acute increase in blood creatinine ≥27 µmol/L or ≥50% within 48 h
**Altered liver parameters:** more than twofold elevation in aminotransferase, alkaline phosphatase, or ɣ-glutamyltransferase
**Hyperammonemia**
mild: 101–200 µmol/L
moderate: 201–300 µmol/L
severe: ≥301 µmol/L
**Anemia:** abnormally low hemoglobin levels

**Table 2:** Baseline characteristics of 131 patients (2 months to 89 years of age) with acquired pyroglutamic acid acidosis. Data are presented as frequency.

Female:male ratio	3.7:1.0
Age	
years	60 [40–76]
≤16 years	10 (7.6)
Pre-existing conditions	
Undernutrition	63 (48)
Kidney disease	26 (20)
Chronic ^†^	23
Acute	3
Alcohol-use disorder	25 (19)
Pregnancy	4 (3.1)
None	34 (26)
Ongoing infection	91 (69)
Acute	83
Chronic (or recurrent)	8
Symptoms and signs	
Diminished level of consciousness	79 (60)
Kussmaul breathing	74 (56)
Nausea or vomiting	36 (27)
Responsible drug	
Paracetamol alone	83 (63)
β-lactamase-resistant penicillin alone ^✢^	4 (3.1)
Paracetamol and penicillin ^✢^	38 (29)
Vigabatrin	3 (2.3)
None of the mentioned drugs *	3 (2.3)
History of recurrences ^‡^	8 (6.1)

^†^ Including two patients on kidney replacement therapy [118,121]; ^✢^ flucloxacillin (n = 38), dicloxacillin (n = 2), cloxacillin (n = 1), naficillin (n = 1); * use of paracetamol not mentioned, but not explicitly excluded in these three cases; ^‡^ one recurrence in 3, two or more recurrences in 5 cases.

**Table 3:** Clinical and laboratory data, therapy and course in 131 patients with acquired pyroglutamic acid metabolic acidosis. Data are presented either as frequency (often with percentage) or as median [with interquartile range]. Parameters with statistically different results between groups are presented in bold.

	All	Paracetamol Alone	Paracetamol and Other Drugs	Further Cases	*p*-Value
N	131	83	38	10	
Female:male ratio	3.7:1.0	4.9:1.0	2.2:1.0	4.0:1.0	0.0756
Age, years	60 [40–76]	55 [34–68]	77 [72–83]	62 [27–76]	<0.0001 *
Clinical and laboratory data					
Pre-existing condition	97 (74)	66 (80)	25 (66)	6 (60)	0.1768
Paracetamol					
For >10 days	84 (64)	58 (70)	26 (68)	NA	0.6708
High dose	15 (11)	14 (17)	1 (2.6)	NA	**0.0353**
Acid–base balance					
pH	7.19 [7.12–7.29]	7.18 [7.09–7.26]	7.24 [7.16–7.33]	7.17 [7.14–7.29]	0.0681
pCO_2_, mm Hg	16 [13–23]	16 [13–23]	18 [12–22]	16 [15–34]	0.7288
Bicarbonate, mmol/L	7 [5–10]	6 [5–10]	8 [5–10]	8 [5–13]	0.3442
Sodium, mmol/L	140 [137–145]	139 [136–143]	143 [138–146]	150 [143–154]	0.0635
Potassium					
mmol/L	3.6 [3.0–4.4]	4.0 [3.4–4.6]	3.0 [2.6–3.7]	3.6 [3.3–3.9]	0.0002 **
<3.5 mmol/L	32 (24)	14 (17)	17 (45)	1 (10)	0.0015 **
Chloride, mmol/L	108 [100–114]	106 [100–111]	114 [102–119]	113 [105–128]	0.0222 **
Anion gap, meq/L	25 [20–30]	27 [21–32]	22 [19–28]	25 [19–32]	0.0489 **
Hyperlactacidemia	13 (10)	11 (13)	2 (5.3)	0	0.4242
mild	7	5	2	0	
moderate	4	4	0	0	
severe	2	2	0	0	
Urinary glycine test positive	28 (21)	20 (24)	8 (21)	0	0.2254
Kidney function deterioration	54 (41)	36 (43)	16 (42)	2 (20)	0.3707
Not requiring replacement therapy	31	20	11	0	0.1251
Requiring replacement therapy	23	16	5	2	
Altered liver parameters ^Δ^	45 (34)	36 (43)	7 (18)	2 (20)	0.0172 ***
Hyperammonemia	9 (6.9)	6 (7.2)	1 (2.6)	2 (20)	**0.1742**
Mild	6	4	1	1	
Moderate	1	1	0	0	
Severe	2	1	0	1	
Anemia	25 (19)	13 (16)	10 (26)	2 (20)	0.4285
Therapy					
Sodium bicarbonate	82 (63)	46 (55)	31 (82)	5 (50)	0.0017 **
Acetylcysteine	55 (42)	36 (43)	18 (47)	1 (10)	0.0918
Course					
Recovery time, days	5 [2– 7]	4 [2–7]	6 [4–7]	4 [2–6]	0.6962
Death	24 (18)	13 (16)	9 (24)	2 (20)	0.5131

* paracetamol and other drugs versus paracetamol alone and further cases; ** paracetamol and other drugs versus paracetamol alone; *** paracetamol alone versus paracetamol and other drugs and further cases; ^Δ^ the elevation was less than 10-fold in all but one case.

**Table 4:** Characteristics of patients with and without acetylcysteine treatment. Data are presented either as frequency (percentage) or as median [interquartile range].

	Acetylcysteine		
	Without	With	*p*-Values
Cases, n	76	55	
Female:male ratio	3.9:1.0	3.6:1.0	>0.9999
Age, years	60 [41–76]	58 [38–77]	0.6970
Pre-existing condition *	55 (72)	42 (76)	0.6884
Baseline blood values			
pH	7.20 [7.14–7.29]	7.19 [7.09–7.29]	0.7446
pCO_2_, mm Hg	15 [14–23]	17 [11–23]	0.7937
Na^+^, mmol/L	140 [136–145]	140 [137–145]	0.9052
K^+^, mmol/L	3.6 [2.9–4.5]	3.7 [3.3–4.3]	0.5663
Bicarbonate, mmol/L	7 [5–10]	6 [4–11]	0.8533
Anion gap, meq/L	28 [24–31]	28 [22–31]	0.5602
Creatinine, µmol/L	144 [88–210]	105 [78–192]	0.0854
Bicarbonate treatment	48 (63)	34 (62)	>0.9999
Recovery time, days	5 [2–7]	5 [2–8]	0.8762
Death	18 (24)	6 (11)	0.0707

* undernutrition, kidney disease, alcohol-use disorder or pregnancy.

**Table 5:** Summary of data from 7 articles addressing the relationship between acid–base balance and pyroglutamic acid.

**Mayatepek et al. (1999)—Germany [122]**
Twenty pediatric patients from a metabolic disease center had a relevantly increased pyroglutamic acid excretion. Fourteen had an inborn error of metabolism, while causes in the other six included paracetamol in one, vigabatrin in another, and unknown factors in four cases.
**Mizock et al. (2004)—United States of America [123]**
Twenty-three critical care unit patients had unexplained gap metabolic acidosis. Four were on paracetamol. All had very low blood levels of pyroglutamic acid.
**Jessurun et al. (2016)—Netherlands [124]**
The Dutch Pharmacovigilance Center annually collects approximately 30,000 reports of adverse drug reactions. It received 12 reports of pyroglutamic metabolic acidosis in female patients (aged 52 to 87, median 72) treated with paracetamol and flucloxacillin, typically occurring 10 days or more after the last medication adjustment.
**Berbee et al. (2017)—Netherlands [73]**
Among 1057 inpatients prescribed both paracetamol and flucloxacillin, 51 had acidosis (pH ≤ 7.35). Only one had an elevated blood anion gap and was diagnosed with secondary pyroglutamic acidosis.
**Gamarra et al. (2019)—Spain [125]**
Twenty-eight critical care patients (22 males, 6 females; average age 62) with septic shock and poor nutrition (average albumin 27 g/L) were studied. Despite normal acid-base balance, they had high levels of pyroglutamic acid and low glutathione in urine and blood.
**Raibman Spector et al. (2019)—Israel [88]**
Thirty-four patients with otherwise unexplained gap metabolic acidosis, not taking paracetamol, were studied. Three had minimally elevated levels of pyroglutamic acid, considered clinically irrelevant. Among six additional patients on paracetamol, one showed a mildly elevated level of pyroglutamic acid.
**Mullins et al. (2020)—Untied States of America [126]**
Among 23 cases of acute paracetamol poisoning, blood levels of pyroglutamic acid were normal (17 cases) or only minimally elevated (6 cases).

## Data Availability

Data sharing is not applicable to this report as no new data were produced during this study.
